# Tumor location as a novel high risk parameter for stage II colorectal cancers

**DOI:** 10.1371/journal.pone.0179910

**Published:** 2017-06-23

**Authors:** Biyuan Wang, Jiao Yang, Shuting Li, Meng Lv, Zheling Chen, Enxiao Li, Min Yi, Jin Yang

**Affiliations:** 1 Department of 1Medical Oncology, First Affiliated Hospital of Xi’an Jiaotong University, Xi’an, Shaanxi Province, China; 2 Breast Surgical Oncology, University of Texas MD Anderson Cancer Center, Houston, TX, United States of America; Institut national de la recherche scientifique, CANADA

## Abstract

Current studies do not accurately evaluate the influence of tumor location on survival of colorectal cancer patients. This study aimed to explore whether tumor location could be identified as another high-risk factor in stage II colorectal cancer by using data identified from the Surveillance, Epidemiology, and End Results database. All colorectal cancer patients between 2004 and 2008 were grouped into three according to tumor location. Of 33,789 patients diagnosed with stage II colorectal cancer, 46.8% were right colon cancer, 37.5% were left colon cancer and 15.7% were rectal cancer. The 5-year cancer specific survivals were examined. Right colon cancer was associated with the female sex, older age (> 50), and having over 12 lymph nodes resected. Conversely, rectal cancer was associated with the male sex, patients younger than 50 years of age and insufficient lymph node resection. The characteristics of left colon cancer were between them and associated with Asian or Pacific Islander populations, T4 stage, and Grade II patients. The prognostic differences between three groups were significant and retained after stratification by T stage, histological grade, number of regional nodes dissected, age at diagnose, race and sex. Furthermore, the significant difference of location was retained as an independent high-risk parameter. Thus, stage II colorectal cancers of different locations have different clinic-pathological features and cancer-specific survivals, and tumor location should be recognized as another high-risk parameter in stage II colorectal cancer.

## Introduction

Colorectal cancer is the third most common malignancy diagnosed in both men and women and is also the third leading cause of cancer deaths in the United States [1]. Extensive efforts have been directed toward early diagnosis and suitable treatment, such as screening, surgical resection, and chemoradiotherapy, in order to provide colorectal cancer patients with personalized approaches. For example, in 2010, the seventh edition of the American Joint Committee on Cancer Staging guidelines subdivided stage II colorectal cancer into IIA (T3N0), IIB (T4aN0), and IIC (T4bN0), which is particularly important for later clinical prognosis and treatment.

According to the NCCN (National Comprehensive Cancer Network) and ESMO (European Society for Medical Oncology) guidelines, adjuvant chemotherapy should be performed for stage II colon cancer patients with clinical high-risk features, including obstruction, perforation, poor/undifferentiated grade, pT4, fewer than 12 lymph nodes resected, lymph or vascular invasion, and resection margin status classified as unclear. The exploration of other prognostic features continues, with the aim to identify these patients and to predict adjuvant therapeutic benefits, including other clinicopathological factors or molecular markers. For example, Quah et al. and Chan et al. [2,3] reported that high levels of preoperative carcinoembryonic antigen was a high-risk feature, and Sinicrope et al. [4] found that dMMR (deficient DNA mismatch repair) was independently associated with improved survival rates.

For decades, many studies have reported epidemiological, clinical, and molecular biological differences between RCC and LCC [5–7]. Regarding the relationship between tumor location and prognosis, there are studies [8–10] showing that patients with RCC (right colon cancer) and those with LCC (left colon cancer) have different outcomes, with few studies focusing on the different survival rates in stage II patients. However, most studies have not defined the differences in survival rates between colon cancers and rectal cancers, and rectal cancer displays its own unique features, with similarities and differences to colon cancers. Rutter et al. [11] and Andreoni et al. [12] reported that patients diagnosed with stage II rectal cancer had a poorer survival rate than stage II colon cancer patients. Fischer et al. [13] reported that 5-year cause-specific and overall survival for stage II–III rectal cancers are as good as those for colon cancer. Thus, there is a need to include patients with ReC (rectal cancer) in studies of early-stage colorectal cancers. Based on our former study [10], which covers the profile of all-stages-colorectal cancers including RCC, LCC and ReC, we observed different outcome characteristics among stages II disease compared with regional and metastatic disease. The result of subgroup-analysis stimulates us to make new explorations regarding clinical decision.

Here, we hypothesized that the outcome of stage II colorectal cancer patients differed with the location of the tumors. To confirm our hypothesis, we examined the relationship between location and 5-year cancer specific survival (CSS) among these patients, as well as other risk features. Our study aimed to provide an overall comparison of stage II patients with ReC, RCC, and LCC.

## Methods

### Data sources

This study was based on data from the Surveillance, Epidemiology, and End Results (SEER) database. The SEER Program is a national database that is funded and maintained by the National Institutes of Health/National Cancer Institute (NIH/NCI). SEER data are publicly available for studies of cancer-based epidemiology and health policy, and thus are exempt from IRB review.

### Patient selection

The SEER*Stat 8.3.2 software was used to identify patients who were diagnosed with stage II colorectal cancer between 2004 and 2008 and were pathologically confirmed as having adenocarcinoma. Patients were excluded if the tumor location and T stage of the colorectal tumor were missing. Age, sex, race, tumor grade, tumor histology, number of regional nodes examined, survival months, cause of death, and vital status, and year of diagnosis were assessed. Based on the registered information of the tumor location, there were three groups: RCC were defined as those tumors arising from the cecum, to and including the transverse colon (C18.0, C18.2, C18.3, C18.4); LCC were defined as those tumors arising from the splenic flexure down to and including the recto-sigmoid junction (C18.5, C18.6, C18.7, C18.9); and ReC (C20.9). Cancer specific survival was calculated as the date of diagnosis to the date of cancer-related death, or the date the patient was last known to be alive. Patients who did not experience these endpoints were described at last follow-up time to December 31, 2013.

### Statistical analyses

The correlations between clinicopathological parameters and locations of colorectal cancers were analyzed by chi-square tests and Kruskal–Wallis tests. Kaplan–Meier survival analyses and Cox regression analyses were used to assess the long-term CSS between RCC, LCC, and ReC with a P-value < 0.05 denoting significance. Analyses were carried out using the Statistical Package for Social Sciences, version 18 (SPSS, Chicago, IL, USA).

## Results

### Patients

Between January 2004 and December 2008, the records of 33,789 patients with stage II colorectal cancer were extracted with a median follow-up time of 60 months (range, 0–107 months) [median age, 72 years (range, 17–104 years)]. The majority of colorectal cancer patients were White population (82.6%), T3 stage (87.6%), grade II (74.1%), with the number of lymph nodes resected over 12 (60.4%), and over 50 years of age (92.3%). Among these patients, subjects having RCC (46.8%) significantly outnumbered those with LCC (37.5%) and ReC (15.7%). (Table 1).

**Table 1:** Demographics and clinical characteristics of stages II colorectal cancer patients stratified by Location.

Characteristic	Overall (N = 33,789)	Right colon cancer (N = 15,816;46.8%)	Left colon cancer (N = 12,683;37.5%)	Rectal cancer (N = 5,290;15.7%)	P value
Age at diagnosis (y)					<0.0001
Median (Range)	72 (17–104)	75(18–103)	70(17–104)	66(19–97)	
<50	2603 (7.7)	852 (5.4)	1127 (8.9)	624 (11.8)	
≥50	31186 (92.3)	14964 (94.6)	11556 (91.1)	4666 (88.2)	
Sex					<0.0001
Male	17196 (50.9)	7258 (45.9)	6786 (53.5)	3152 (59.6)	
Female	16593 (49.1)	8558 (54.1)	5897 (46.5)	2138 (40.4)	
Race					<0.0001
A/PI	2428 (7.2)	850 (5.4)	1124 (8.9)	454 (8.6)	
White	27924 (82.6)	13300 (84.1)	10211 (80.5)	4413 (83.4)	
Black	3437 (10.2)	1666 (10.5)	1348 (10.6)	423 (8.0)	
T stage					<0.0001
T3	29590 (87.6)	13990 (88.5)	10928 (86.2)	4672 (88.3)	
T4	4199 (12.4)	1826 (11.5)	1755 (13.8)	618 (11.7)	
Tumor grade					<0.0001
I	2286 (6.8)	1071 (6.8)	858 (6.8)	359,6.7(15.6)	
II	25023 (74.1)	11122 (70.3)	10097 (79.6)	3804 (71.9)	
III &IV	5614 (16.6)	3412 (21.6)	1499 (11.8)	703 (13.3)	
Unknown	866 (2.6)	211 (1.3)	229 (1.8)	426 (8.1)	
No. of lymph nodes examined					<0.0001
≥12	20422 (60.4)	11166 (70.6)	7231 (57.0)	2025 (38.3)	
<12	13078 (38.7)	4534 (28.7)	5343 (42.1)	3201 (60.5)	
Unknown	289 (0.9)	116 (0.7)	109 (0.9)	64 (1.2)	

Abbreviations: A/PI, Asian or Pacific Islander

### Clinicopathological characteristics

The median ages at diagnosis of ReC patients and LCC patients were younger than that of RCC patients (66, 70, and 75 years of age, respectively). The percentages of male patients were increased with RCC, LCC, and ReC (45.9%, 53.5%, and 59.6%, respectively). RCC patients were more likely to be White than LCC and ReC patients (84.1%, 80.5%, and 83.4%, respectively). Subjects with T4 stage tumors were greater in the LCC patients than in the RCC and ReC patients (13.8%, 11.5%, and 11.7%, respectively). Histologically, the RCC tumors were of higher grade than the LCC and ReC tumors, with 21.6% of the RCC tumors being grade III and IV vs. 11.8% of the LCC tumors, and 13.3% of the ReC tumors. The percentages of patients with less than 12 lymph nodes dissected in the LCC and ReC patients were larger than that of the RCC patients (60.5%, 42.1% and 28.7%, respectively). Similar conclusions can be obtained from the S1 Table.

### Overall CSS as classified by tumor location

**Fig 1:**
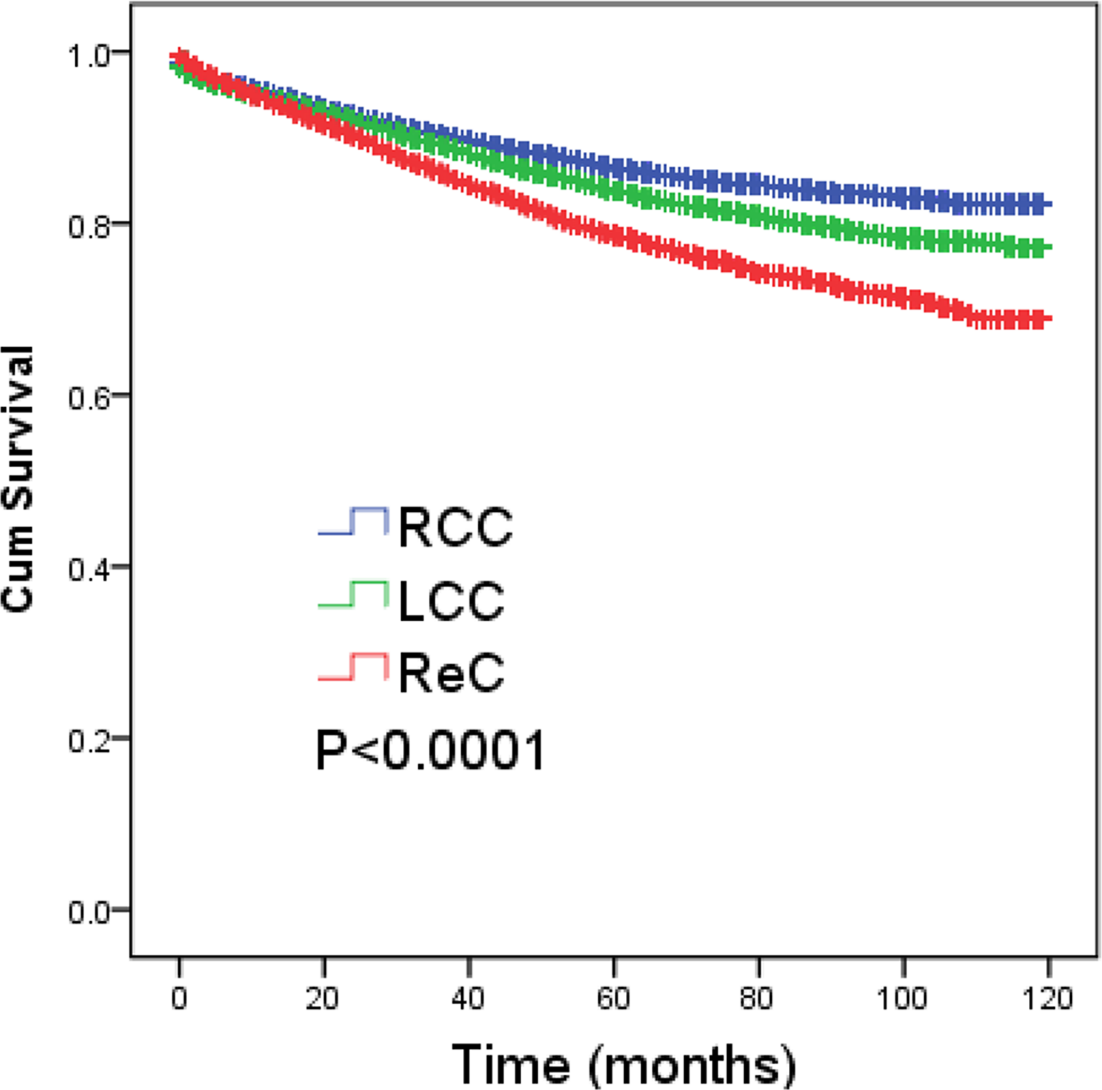
Cancer-specific survival curves for patients with RCC, LCC, and ReC. Kaplan–Meier curves showing the comparisons of disease-specific survival among right colon cancer (RCC), left colon cancer (LCC) and rectal cancer (ReC) with a significant difference (P<0.0001).

Using univariate survival analyses, the tumor location was shown to be a significant factor (P < 0.001) that affected 5-year CSS (Fig 1). Five year CSS percentages were 86.5%, 83.8%, and 78.7% among patients with RCC, LCC, and ReC, respectively. T stage, histological grade, regional nodes dissected, age at diagnosis, and race also influenced survival (data not shown). Similarly, the survival curves for stages II colorectal cancer patients stratified by six sites have a significant difference as S1 Fig showed, which was in accord with the survival analyses of three locations.

### Comparisons in stratified analyses

In the stratified survival analyses of stage II colorectal cancers with factors including T stage (T3 vs. T4), histological grade (Grade I vs. Grade II vs. Grade III and IV), number of regional nodes dissected (≥ 12 vs. < 12), age at diagnosis (< 50 years of age vs. ≥ 50 years of age), race/ethnicity (Asian or Pacific Islander vs. White vs. Black), sex (male vs. female), significantly different outcomes between RCC, LCC, and ReC were found in all groups (S2–S7 Figs). Moreover, it is interesting to note that the two curves for RCC and LCC did not show a prognostic difference in stratified survival analyses for Asians or Pacific Islanders, while both had gaps between ReC.

In addition, the paired stratified survival analyses between any two of the three locations presented similar results, i.e., most groups with six factors were statistically significant (Table 2). Consistent with the results of the survival curves, the stratified survival analyses for Asian or Pacific Islander patients with LCC and RCC were not significant (P = 0.9647). In addition, the stratified analyses of Grade I patients with LCC and RCC showed no statistical significance, perhaps due to the better outcome of the groups.

**Table 2:** The paired stratified survival analyses between two locations in stage II colorectal cancer patients.

Factors		RCC vs REC		LCC vs REC		LCC vs RCC	
		HR	P-value	HR	P-value	HR	P-value
Sex							
Female		1.369	< .0001	1.477	< .0001	1.276	< .0001
		(1.301–1.441)		(1.331–1.640)		(1.174–1.386)	
Male		1.255	< .0001	1.288	< .0001	1.224	< .0001
		(1.195–1.318)		(1.171–1.417)		(1.124–1.334)	
Age at diagnosis(y)							
<50		1.51	< .0001	1.697	< .0001	1.342	0.0343
		(1.314–1.735)		(1.337–2.154)		(1.021–1.763)	
≥50		1.305	< .0001	1.351	< .0001	1.264	< .0001
		(1.258–1.354)		(1.255–1.455)		(1.189–1.344)	
Race							
A/PI		1.273	0.0006	1.622	0.0003	1.006	0.9647
		(1.107–1.464)		(1.247–2.110)		(0.782–1.293)	
White		1.31	< .0001	1.363	< .0001	1.262	< .0001
		(1.260–1.361)		(1.261–1.474)		(1.181–1.348)	
Black		1.333	< .0001	1.305	0.0137	1.369	0.0001
		(1.197–1.484)		(1.055–1.614)		(1.165–1.610)	
T stage							
T3		1.308	< .0001	1.455	< .0001	1.18	< .0001
		(1.257–1.361)		(1.343–1.577)		(1.102–1.264)	
T4		1.3	< .0001	1.277	0.0011	1.332	< .0001
		(1.204–1.404)		(1.101–1.481)		(1.181–1.502)	
Tumor grade							
I		1.35	< .0001	1.61	0.0008	1.144	0.2802
		(1.175–1.551)		(1.216–2.131)		(0.896–1.461)	
II		1.285	< .0001	1.312	< .0001	1.259	< .0001
		(1.231–1.340)		(1.207–1.425)		(1.174–1.350)	
III&IV		1.365	< .0001	1.454	0.0001	1.301	0.0004
		(1.254–1.485)		(1.208–1.750)		(1.125–1.504)	
No. of lymph nodes examined							
≥12		1.154	< .0001	1.166	0.0147	1.141	0.0015
		(1.086–1.225)		(1.030–1.319)		(1.052–1.239)	
<12		1.21	< .0001	1.276	< .0001	1.153	0.0019
		(1.153–1.269)		(1.167–1.394)		(1.054–1.262)	

Abbreviations: CI confidence interval, HR hazard ratio, A/PI, Asian or Pacific Islander, RCC, right colon cancer, LCC, left colon cancer, ReC, rectal cancer

### Multivariate survival analyses

Tumor location as an independent prognostic factor was retained in multivariable analyses (P < 0.001), along with age at diagnosis, race/ethnicity, T stage, tumor histological grade, and number of regional nodes dissected in all stage II colorectal cancer patients (Table 3). The prognosis of RCC was the best in the three groups, the prognosis of ReC was the worst, and the prognosis of LCC was between RCC and ReC. More detailed data of multivariate analyses of stage II colorectal cancer patients by six sites can be found in S2 Table.

**Table 3:** Multivariate analyses of stage II colorectal cancer patients.

Factors	P-value	HR(95%CI)
Location	0.000	—
RCC	0.000	0.642(0.595–0.692)
LCC	0.000	0.760(0.706–0.819)
Grade	0.000	—
I	0.000	0.784(0.693–0.888)
II	0.000	0.867(0.807–0.930)
T-Stage	0.000	2.651(2.486–2.827)
No. of regional nodes examined	0.000	1.567(1.487–1.651)
Race	0.000	—
A/PI	0.000	0.604(0.529–0.689)
White	0.000	0.758(0.698–0.822)
Age at diagnosis	0.000	1.570(1.404–1.755)

Abbreviations: RCC, right colon cancer, LCC, left colon cancer, ReC, rectal cancer, A/PI, Asian or Pacific Islander; 95% CI, 95% confidence interval; HR hazard ratio

## Discussion

All clinicopathological characteristics, including sex, age at diagnosis, race/ethnicity, T stage, tumor histological grade, and number of regional nodes dissected, were analyzed and shown to have a significant correlation with tumor location. The stage II RCCs were more frequent in females, in patients over 50 years of age, and were characterized with adequate lymph nodes being resected, while younger, male patients with insufficient lymph node resection cancers were more common in stage II ReC patients. LCC occurrence was between the other types in numbers, except for the highest proportion of T4 stage tumors. The survival analyses showed that the more proximal the cancer was located, the better outcome it had. Tumor location was recognized as an independent prognostic feature in multivariable analyses. These findings demonstrated the prognostic value of tumor location in stage II colorectal cancer patients. At the same time, the prognostic effect was retained and consistent in the supplementary analysis of six sites (i.e. cecum, ascending colon, transverse colon, descending colon, sigmoid colon and rectum), and the cecum colon was specific due to the highest proportion of T4 stage tumors.

Importantly, the survival curves of stage II RCC and LCC were similar in Asian or Pacific Islander patients, suggesting that there is an absolute survival difference between colon cancers and rectal cancers. Our analyses also confirmed that examined nodal status, histologic grade, and depth of tumor invasion into the bowel wall (T3 to T4) were significant prognostic factors in stage II colorectal cancers.

Weiss et al. [8] reported that stage II right-sided cancers had lower mortality percentages than did left-sided cancers [hazard ratio (HR), 0.92; 95% confidence interval (CI), 0.87–0.97; P < 0.001], while no significant difference was observed in mortality percentages between right- and left-sided cancers for all stages (HR, 1.01; 95% CI, 0.98–1.04; P < 0.598), or for stage I cancers (HR, 0.95; 95% CI, 0.88–1.03; P < 0.211). Likewise, according to Meguid et al. [9], among subjects with stage II tumors, those with right-sided tumors had a far better survival outcome when compared with that of left-sided tumors (HR 0.91; P < 0.001), in contrast to those of other subgroups. These results are consistent with our findings, and also support the right and left side classification model [10]. Notably, their inclusion and exclusion criteria differed. For example, they excluded patients who had rectal cancers. Although these studies were also population-based, our patients were diagnosed at a date later than 2004–2008, and they presented a condition closer to that currently used in clinical practice.

Among the stage II colon cancer patients, there is ongoing research on the identification of the subgroups that could benefit from adjuvant chemotherapy. However, studies of the advantages of adjuvant chemotherapy [14,15], clinical evidence concerning improved prognosis in the high-risk groups [16,17], and studies of clinical efficacy and patient selection for adjuvant chemotherapy [18–20] were designed to maximize the survival benefits to “high-risk” patients. Our studies helped to define this subgroup of patients. In our study, we clearly demonstrated that the location of colon cancer tumors should be included as a high-risk factor and as a factor in decisions concerning treatments of some colon cancers.

However, in contrast to some colon cancers, rectal carcinomas have a high risk of local recurrence, especially for locally advanced rectal cancers. This risk is associated with the close proximity of the rectum to pelvic structures and organs and to the technical difficulties associated surgical margins at resection. Therefore, in the NCCN clinical practice guidelines, the combination of chemotherapy and radiotherapy before surgical resection with total mesorectal excision (TME), as well as postoperative chemotherapy, are recommended to improve both local control and overall survival [21,22]. Considering the worse outcome of ReC patients in our analyses, as well as the worse patient survival found in studies other than in colon cancers [11,23,24], the multimodality therapy still needs to be further explored for rectal cancer patients.

Regarding the genetic mechanisms, there are potential explanations for our findings. For example, based on 3,045 stage II colon cancer patients who received adjuvant chemotherapy, Missiaglia et al. [25] found that RCC patients relapsed significantly less frequently than LCC patients, and the microsatellite instability status was thought to be the cause. Due to somatic inactivation of the DNA mismatch repair (MMR) genes, microsatellite instability (MSI) was discovered in approximately 15% of colon cancer samples [26]. In related studies, MSI-H tumors were more often characterized as stage II [27–29], and were more often found in RCC, compared to in LCC and ReC [30–32]. Several retrospective studies [33,34], a meta-analysis [35], and recent large population clinical trials [4,36–38] support that MSI-H colon cancer patients have a better survival than those with MSI-L/S tumors. Furthermore, MSI-H patients do not benefit from 5-fluorouracil (FU)-based adjuvant chemotherapy [34,37,39]. However, due to the lack of MSI status and adjuvant chemotherapy information, it is difficult to judge whether the better outcome for RCC was related with MSI-H status.

Except microsatellite instability, tumor markers which reflect the heterogeneity of CRC include: the CpG island methylator phenotype (CIMP), as well as somatic point mutations in BRAF and KRAS, and other molecular genetic markers, TP53 and PIK3CA for example[40–42]. First, gene silence resulted by methylation of particular CpG islands can be a driver of carcinogenesis [43]. Considering the tumor location, CIMP-positive CRC account approximately 30–40% of RCC and 5–15% of LCC and ReC [44]. However, studies showed that the association between CIMP status and survival of CRC was inconsistent [45,46]. Second, mutations status of KRAS, NRAS, BRAF genes were used as predictive markers to anti-EGFR therapy. Moreover, there was large-scale study which demonstrated the association of BRAF mutations with localization of RCC [28].However, their prognostic values were still uncertain [28,47,48], especially in early CRC. In conclusion, many studies suggested systems for CRC classification [49–51], but it’s not easy to summarize the diversity and complexity of CRC, only use the above markers, which indeed included the associations between protein expression patterns and genomic and epigenomic characteristics of CRC. Considering the diversity of possible scenarios in CRC development, including different start points, key pathways and a number of bifurcation points during the disease progression, we highly advocate widening the further investigations by including the location of CRC to analyze molecular subtypes in the future.

Our study is one of the largest population-based studies designed to show the survival comparison on the basis of sufficient follow-up time in a three-part model. We demonstrated a lower cancer-specific mortality for proximal colon cancers compared with distal colon cancers in stage II disease and the significant prognostic value of traditional two-colon model. More than that, we explored and showed a similar but not completely consistent result in continuum model in supplementary information. Except the poor prognosis of cecum cancers in multivariate analyses and cancer-specific survival curves, the risk of mortality contributed by colorectal cancers was increased through bowel subsites. Recent reports observed the gradually change along the length of the colorectum in terms of molecular features [52–54] and pathological features [55], while age, tumor grade and histological subtype support the right and left side classification model. At the same time, Mai Yamauchi et al. found cecum cancers have a potential unique molecular phenotype [54] in continuum model. Our study and these findings indicated that further studies at subsite level are needed to provide additional prognostic information in both models to improve personalized therapeutic strategies.

There are some limitations in our study. First, the SEER database lacked detailed clinical information; therefore we were unable to include the related information concerning adjuvant chemotherapy as well as some specific pathological features like vascular invasion, tumor margin status, and lymphocytic invasion, which reported to be no significant survival differences among RCC, LCC, and ReC patients [30]. Secondly, the study was also limited by its retrospective nature. Thirdly, lack of molecular data limited the analyses on molecular level.

In conclusion, considering the worse outcomes of LCC and ReC patients, when compared with RCC patients, and considering the current treatment for ReC, we suggest that tumor location should be considered as a significant risk factor, in conjunction with the presently used high-risk factors, when considering chemotherapy for postoperative stage II colon cancer patients. A more aggressive intervention for LCC patients is recommended, especially for patients with other high-risk factors. Further studies concerning the benefits of chemotherapy for RCC patients are needed. With the addition of tumor location as a high-risk factor, patients identified as having high-risk should be considered for adjuvant chemotherapy and/or enrollment in investigational clinical trials. Moreover, molecular changes at subsite level help us to understand how location matters in the process of CRC initiates and progresses.

## Supporting information

S1 Table Demographics and clinical characteristics of stages II colorectal cancer patients stratified by sites. (DOCX)

S2 Table Multivariate analyses of stage II colorectal cancer patients by sites. (DOCX)

S1 Fig Cancer-specific survival curves for patients with stages II colorectal cancer patients stratified by sites. (DOCX)

S2 Fig Stratified cancer-specific survival curves for patients with RCC, LCC and ReC by age. (DOCX)

S3 Fig Stratified cancer-specific survival curves for patients with RCC, LCC and ReC by race. (DOCX)

S4 Fig Stratified cancer-specific survival curves for patients with RCC, LCC and ReC by T stage. (DOCX)

S5 Fig Stratified cancer-specific survival curves for patients with RCC, LCC and ReC by histological grade. (DOCX)

S6 Fig Stratified cancer-specific survival curves for patients with RCC, LCC and ReC by number of lymph nodes dissected. (DOCX)

S7 Fig Stratified cancer-specific survival curves for patients with RCC, LCC and ReC by sex. (DOCX)

## Abbreviations

RCC: right colon cancer
LCC: left colon cancer
ReC: rectal cancer
