# Supplementary material for: Tumor location as a novel high risk parameter for stage II colorectal cancers
Source: PLoS One. 2017 Jun 23;12(6):e0179910. doi: 10.1371/journal.pone.0179910 (PMC5482466; doi:10.1371/journal.pone.0179910)
Supplement: S1 Table — (DOCX) [file pone.0179910.s001.docx]

Table S1. Demographics and clinical characteristics of stage II colorectal cancer patients stratified by sites.

|  | Overall  (N=33,789) | cecum  (N=5628;16.7%) | ascending colon  (N=5522;16.3%) | hepatic colon  (N=1664; 4.9%) | transverse colon  (N=3002; 8.9%) | splenic flexure  (N=1207; 3.6%) | descending colon  (N=1752; 5.2%) | sigmoid  (N =7013; 20.8%) | recto-sigmoid junction  (N=2711; 8.0%) | rectum  (N=5290; 15.7%) |
| --- | --- | --- | --- | --- | --- | --- | --- | --- | --- | --- |
| **Sex** | | | | | | | | | | |
| female | 49.1 | 56.8 | 53.7 | 49.3 | 52.5 | 43.8 | 47.5 | 47.8 | 43.6 | 40.4 |
| male | 50.9 | 43.2 | 46.3 | 50.7 | 47.5 | 56.2 | 52.5 | 52.2 | 56.4 | 59.6 |
| **Grade** | | | | | | | | | | |
| I | 6.8 | 6.9 | 6.6 | 7.4 | 6.6 | 6.4 | 6.2 | 6.9 | 7.0 | 6.7 |
| II | 74.1 | 69.9 | 69.5 | 69.3 | 73.1 | 75.7 | 78.7 | 80.7 | 79.2 | 71.9 |
| III&IV | 16.6 | 21.8 | 22.8 | 21.5 | 19.0 | 15.9 | 13.9 | 10.9 | 11.2 | 13.3 |
| **Stage** | | | | | | | | | | |
| T3 | 87.6 | 85.1 | 91.9 | 90.0 | 87.6 | 87.2 | 87.1 | 85.5 | 86.7 | 88.3 |
| T4 | 12.4 | 14.9 | 8.1 | 10.0 | 12.4 | 12.8 | 12.9 | 14.5 | 13.3 | 11.7 |
| **Race** | | | | | | | | | | |
| A/PI | 7.2 | 4.2 | 5.9 | 6.7 | 5.8 | 6.7 | 9.4 | 9.1 | 8.8 | 8.6 |
| White | 82.6 | 84.8 | 84.5 | 83.4 | 82.4 | 78.5 | 77.2 | 81.3 | 81.3 | 83.4 |
| Black | 10.2 | 11.0 | 9.6 | 9.9 | 11.8 | 14.7 | 13.4 | 9.5 | 9.8 | 8.0 |
| **Age** | | | | | | | | | | |
| ＜50 | 7.7 | 5.2 | 4.6 | 6.9 | 6.3 | 6.6 | 9.5 | 8.4 | 10.7 | 11.8 |
| ≥50 | 92.3 | 94.8 | 95.4 | 93.1 | 93.7 | 93.4 | 90.5 | 91.6 | 89.3 | 88.2 |
| **No. of lymph nodes examined** | | | | | | | | | | |
| ≥12 | 60.4 | 71.9 | 73.9 | 70.5 | 62.2 | 58.9 | 59.1 | 56.4 | 56.4 | 38.3 |
| ＜12 | 38.7 | 27.5 | 25.4 | 28.5 | 37.0 | 40.3 | 40.2 | 42.7 | 42.7 | 60.5 |

Abbreviations: A/PI, Asian or Pacific Islander
