# Supplementary material for: Tumor location as a novel high risk parameter for stage II colorectal cancers
Source: PLoS One. 2017 Jun 23;12(6):e0179910. doi: 10.1371/journal.pone.0179910 (PMC5482466; doi:10.1371/journal.pone.0179910)
Supplement: S2 Table — (DOCX) [file pone.0179910.s002.docx]

Table S2. Multivariate analyses of stage II colorectal cancer patients by sites.

| Factors | P-value | HR(95%CI) |
| --- | --- | --- |
| Location | 0.000 | — |
| cecum | 0.000 | 0.687（0.626-0.753） |
| ascending colon | 0.000 | 0.607（0.550-0.670） |
| transverse colon | 0.000 | 0.652（0.582-0.730） |
| descending colon | 0.000 | 0.730（0.641-0.831） |
| sigmoid colon | 0.000 | 0.777（0.716-0.843） |
| Grade | 0.000 | — |
| I | 0.000 | 0.666 (0.554-0.801） |
| II | 0.000 | 0.714 (0.616-0.827) |
| T-Stage | 0.000 | 2.646 (2.471-2.834) |
| No. of Regional nodes examined | 0.000 | 1.555 (1.470-1.646) |
| Race | 0.000 | — |
| A/PI | 0.000 | 0.601 (0.521-0.694) |
| White | 0.000 | 0.759 (0.695-0.829) |
| Age at diagnosis | 0.000 | 1.532 (1.360-1.725) |

Abbreviations: A/PI, Asian or Pacific Islander
