# Supplementary figures and images for: Tumor location as a novel high risk parameter for stage II colorectal cancers
Source: PLoS One. 2017 Jun 23;12(6):e0179910. doi: 10.1371/journal.pone.0179910 (PMC5482466; doi:10.1371/journal.pone.0179910)

S2 Fig. Stratified cancer-specific survival curves for patients with RCC, LCC and ReC by age.


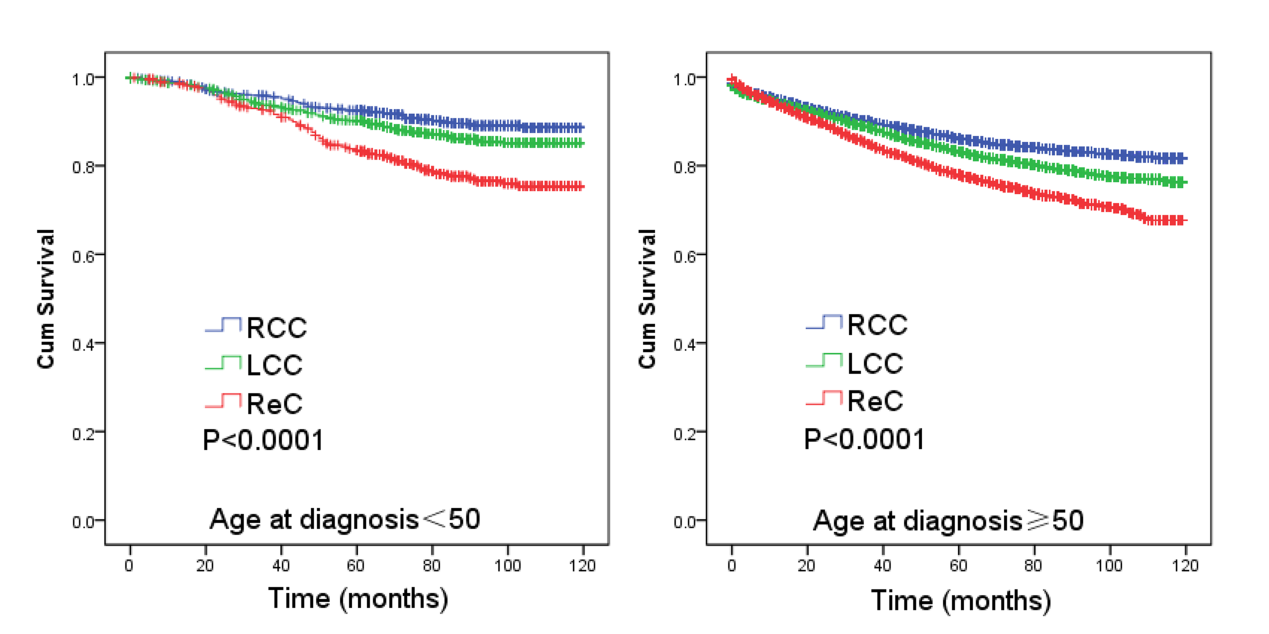

Supplement: S2 Fig — (DOCX) [file pone.0179910.s004.docx]

S3 Fig. Stratified cancer-specific survival curves for patients with RCC, LCC and ReC by race.


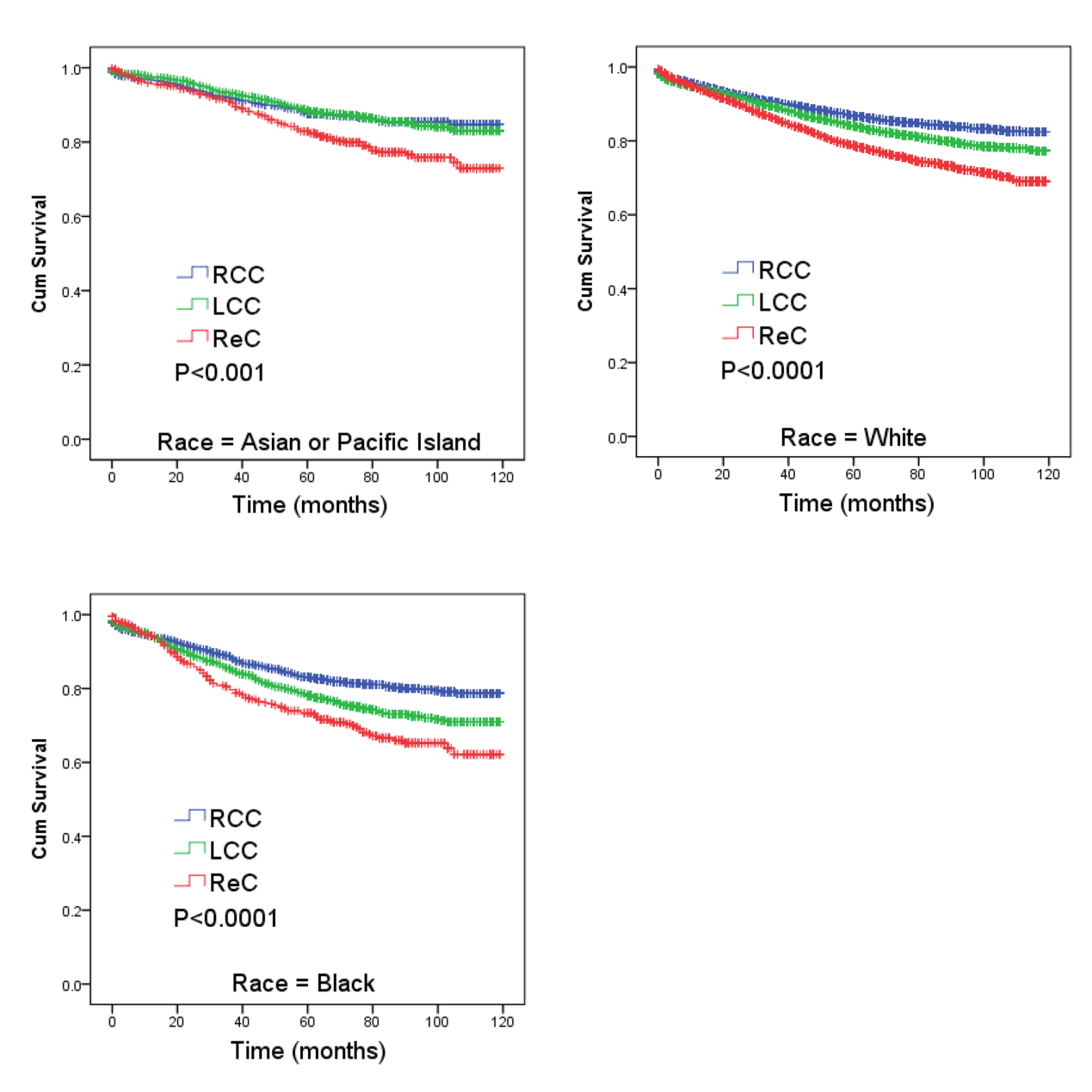

Supplement: S3 Fig — (DOCX) [file pone.0179910.s005.docx]

S4 Fig. Stratified cancer-specific survival curves for patients with RCC, LCC and ReC by T stage.


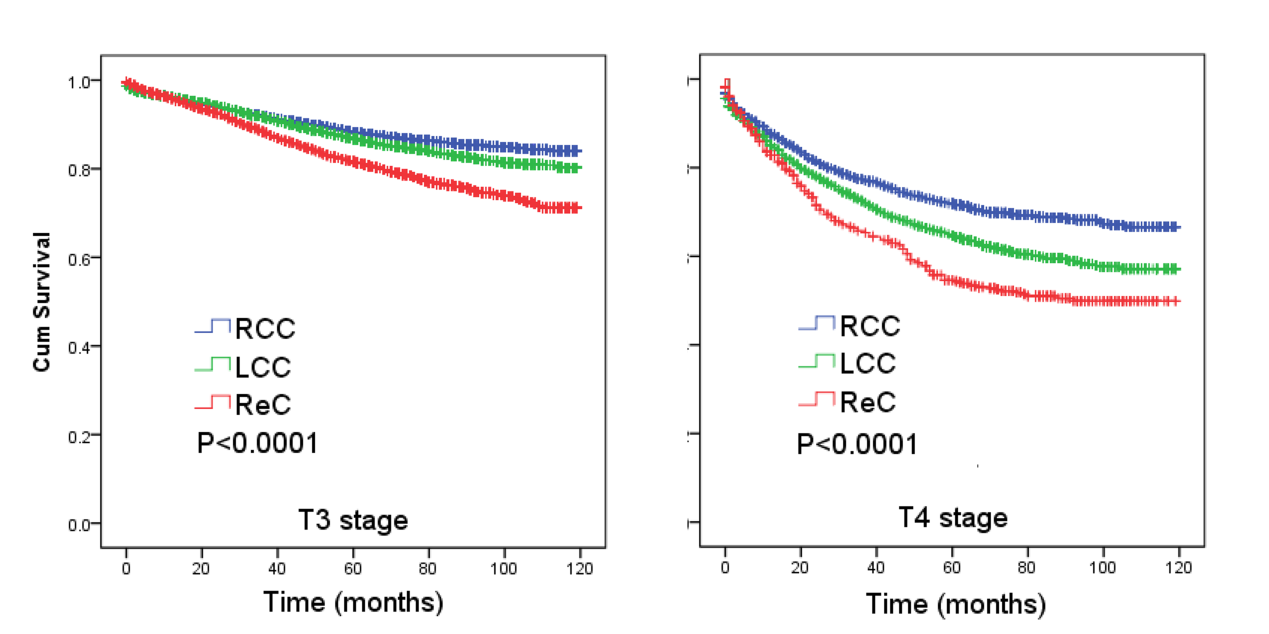

Supplement: S4 Fig — (DOCX) [file pone.0179910.s006.docx]

S7 Fig. Stratified cancer-specific survival curves for patients with RCC, LCC and ReC by sex.


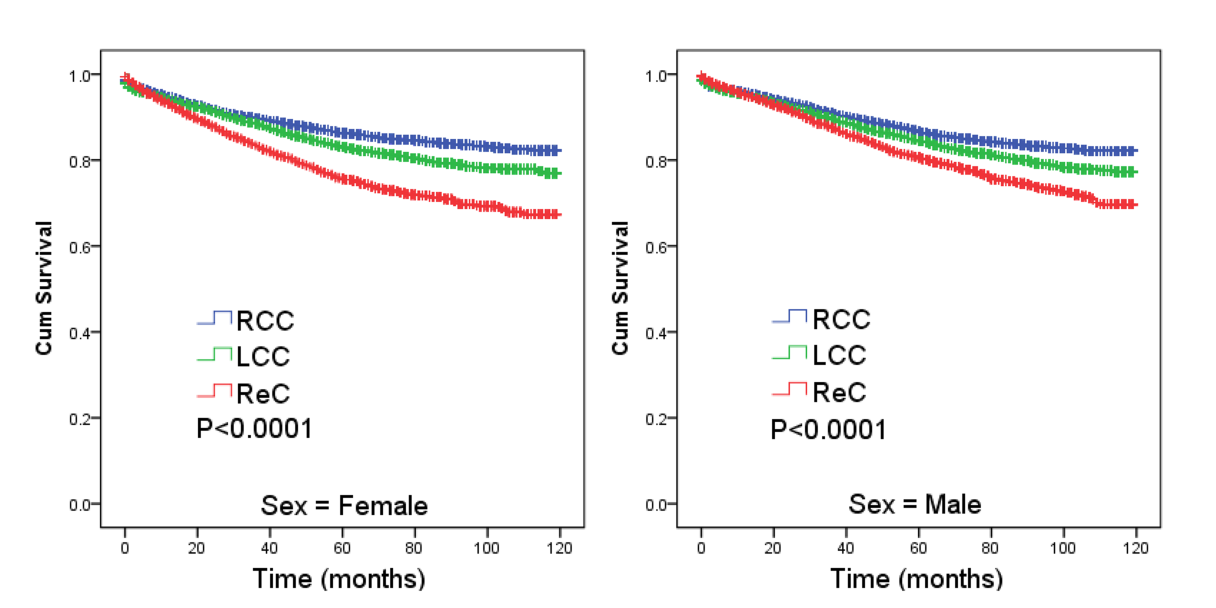

Supplement: S7 Fig — (DOCX) [file pone.0179910.s009.docx]
