# Supplementary material for: Tumor location as a novel high risk parameter for stage II colorectal cancers
Source: PLoS One. 2017 Jun 23;12(6):e0179910. doi: 10.1371/journal.pone.0179910 (PMC5482466; doi:10.1371/journal.pone.0179910)

S5 Fig. Stratified cancer-specific survival curves for patients with RCC, LCC and ReC by histological grade.


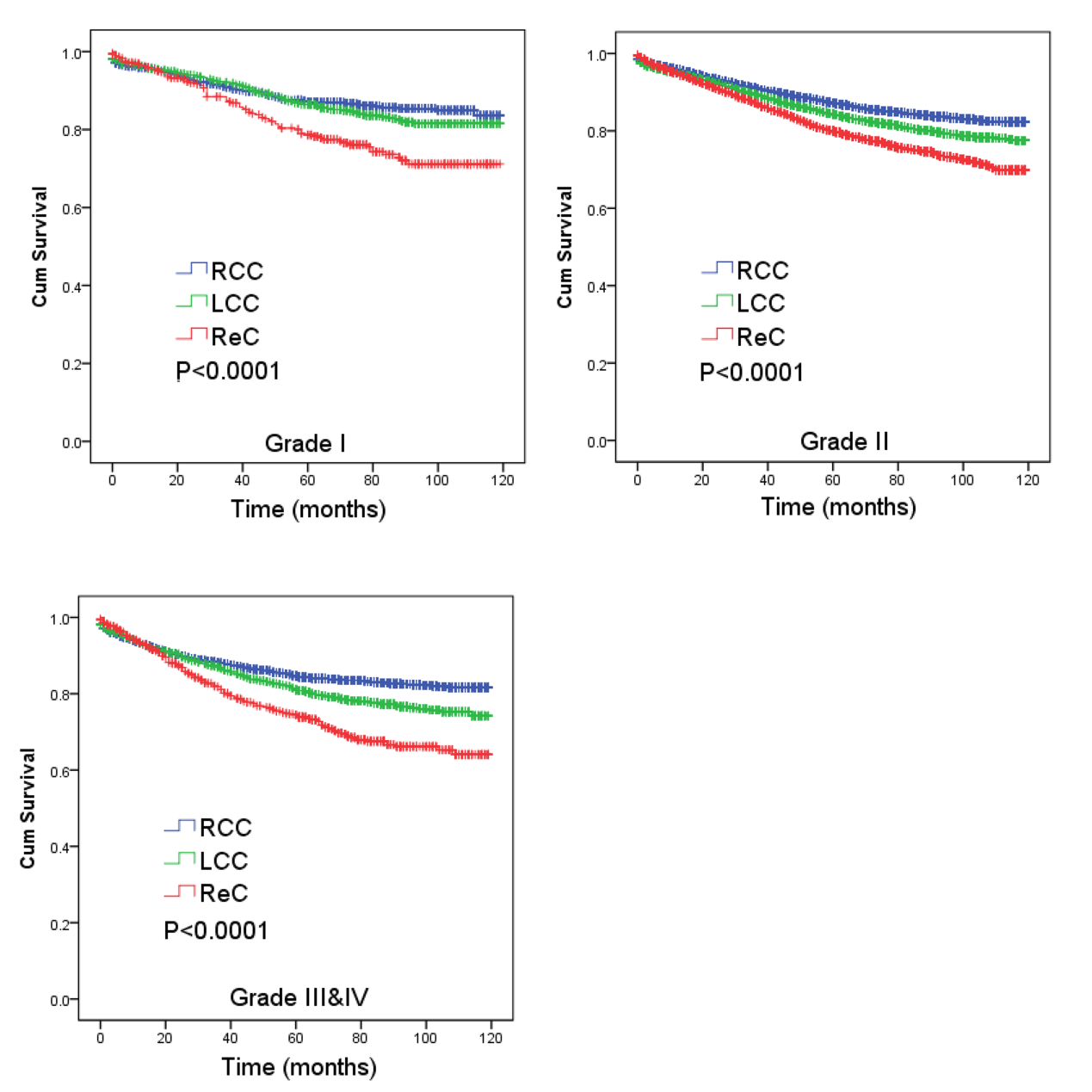

Supplement: S5 Fig — (DOCX) [file pone.0179910.s007.docx]
